# MiR-125b inhibits cardiomyocyte apoptosis by targeting BAK1 in heart failure

**DOI:** 10.1186/s10020-021-00328-w

**Published:** 2021-07-08

**Authors:** Bei Zhang, Shanyong Mao, Xingde Liu, Sha Li, Haiyan Zhou, Ying Gu, Wupeng Liu, Lei Fu, Chunyan Liao, Pengzhen Wang

**Affiliations:** 1 Guizhou Medical University, No. 9 Beijing Road, Yunyan District, Guiyang, Guizhou 550004 People’s Republic of China; 2 Department of Ultrasound Medicine, The Affiliated Hospital of Guizhou Medical University, No. 28 Guiyi Street, Yunyan District, Guiyang, Guizhou 550004 People’s Republic of China; 3 Department of Cardiology, The Second Affiliated Hospital of Guizhou University of Traditional Chinese Medicine, Guiyang, Guizhou 550004 People’s Republic of China; 4 Departmentof Clinical Research Centre, The Affiliated Hospital of Guizhou Medical University, Guiyang, Guizhou 550004 People’s Republic of China; 5 Department of Cardiology, The Affiliated Baiyun Hospital of Guizhou Medical University, Guiyang City, Guizhou 550014 People’s Republic of China

**Keywords:** MiR-125b, BAK1, Heart failure, Apoptosis

## Abstract

**Background:**

Although miR-125b plays a crucial role in many human cancers. However, its function in heart failure (HF) remains unclear. Our study aimed to investigate its involvement in heart failure.

**Methods:**

In this study, the mouse HF model was successfully constructed through transverse aortic constriction (TAC) operation. Changes in mRNA and protein levels in isolated myocytes and heart tissues were examined using qRT-PCR, Western blot and Immunohistochemical staining and immunofluorescent staining. Changes in cardiac functions were examined using ultrasound. Interactions between miR-125b and BAK1 was analyzed using the luciferase reporter assay. Cardiomyocyte apoptosis was evaluated using the TUNEL staining.

**Results:**

We found that miR-125b expression was significantly downregulated in myocardial tissues of HF mice. Moreover, miR-125b upregulation in HF mice injected with agomir-125b efficiently ameliorated cardiac function. Further, miR-125b upregulation significantly decreased the protein levels of apoptosis-related makers c-caspase 3 and Bax, while increased Bcl-2 expression. In addition, BAK1 was identified as a direct target of miR-125b. As expected, BAK1 overexpression observably reversed the effect of agomir-125b on cardiac function and on the expression of apoptosis-related makers in the heart tissues of HF mice.

**Conclusions:**

Taken together, miR-125b overexpression efficiently attenuated cardiac function injury of HF mice by targeting BAK1 through inhibiting cardiomyocyte apoptosis, suggesting that miR-125b/BAK1 axis might be a potential target for the diagnosis or treatment of HF.

**Supplementary Information:**

The online version contains supplementary material available at 10.1186/s10020-021-00328-w.

## Introduction

Heart failure (HF) has become an epidemic disease affecting approximately 1% to 2% of the adult population worldwide (Tanai and Frantz 2015). HF is a complex disease and may occur due to many reasons including high blood pressure, aging, diabetes, coronary heart diseases, and obesity (Lakatta 2002; Burchfield et al. 2013). In the last decades, several treatments such as pacing and defibrillation therapies, heart transplantation or mechanically assisted circulatory support have been widely applied for HF (Abdo 2017; Martinelli et al. 2011). However, HF is still common in adults accounting for substantial morbidity and mortality worldwide, and its prevalence is increasing (Metra and Teerlink 2017). Advances in the molecular mechanisms underlying the HF pathophysiology and treatment have led to the decreased incidence of patients with HF and the quality of life of the patients (Mazurek and Jessup 2017). Therefore, better understanding the specific mechanisms involved in HF will contribute to identifying new targets and even extend the window of treatment for HF.

MicroRNAs (miRNAs) are a group of small RNA molecules with approximately 22 nucleotides in length and play crucial roles in gene silencing and translational repression through directly binding to mRNAs of targets in human diseases (Vishnoi and Rani 2017; Batool et al. 2018; Zhou et al. 2016). Recently, many miRNAs are increasingly recognized to play essential roles in cardiovascular diseases including heart failure, specifically cardiac fibrosis and cardiomyocyte apoptosis (Vegter et al. 2016). MiR-125b has been identified to be closely associated with various biological processes in multiple diseases like nasopharyngeal carcinoma, acute myeloid leukemia and systemic lupus erythematosus (Li et al. 2017; Liu et al. 2017; Cao et al. 2018 Nagpal et al. found that miR-125b is essential for the transition from fibroblast to myofibroblast and cardiac fibrosis (Nagpal et al. 2016; Bie et al. 2016). However, to our best knowledge, its function and specific mechanisms in HF remain unclear.

Bcl-2 homologous antagonist/killer (BAK1), a pro-apoptotic factor, has been identified to play crucial roles in the apoptotic process of human cancer cells or mitochondrial cells in response to various stimulations. For example, BAK1 overexpression significantly induced mitochondrial apoptosis, while 4,4'-diisothiocyanostilbene-2,2'-disulfonic acid (DIDS) inhibited BAK1 overexpression-induced apoptosis via GSK3β/β-catenin signaling pathway (Yang et al. 2018). Costunolide induces mitochondria-mediated apoptosis in human gastric adenocarcinoma BGC-823 cells through upregulating BAK1 expression (Yan et al. 2019). In addition, BAK1 has shown essential functions in cardiovascular diseases such as acute myocardial infarction. miRNA-125-5p could protect the heart against acute myocardial infarction by inhibiting the expression of pro-apoptotic factors BAK1 and KLF13 in cardiomyocytes (Bayoumi et al. 2018a). However, the underlying regulatory network of BAK1 in HF has not been studied in detail.

In this study, we first established the HF mouse model through TAC operation and found that miR-125b was significantly downregulated in heart tissues of HF mice. Further, a series of in vitro and in vivo experiments all confirmed that miR-125b overexpression efficiently alleviate cardiac functional injury in HF mice through directly targeting BAK1, thereby suppressing cardiomyocyte apoptosis, and suggested that miR-125b/BAK1 axis might be a potential target for the diagnosis and treatment of HF.

## Materials and methods

### Animal model

A total of 40 C57BL/6 mice (male, 8–10 weeks old, approximately 22–25 g) were obtained from Guizhou Medical University and kept at 20 ± 2℃ in a room with 12 h light/dark cycle and approximately 50–60% humidity. To construct HF mouse model, mice were fasted for 12 h, depilated, anesthetized with 4% chloral hydrate (0.1 ml/10 g), and subjected to transverse aortic constriction (TAC) operation as previously described (Li et al. 2018a). Briefly, following intubation and mechanical ventilation of the mice, the transverse aorta was accessed through partial thoracotomy via the upper edge of the sternum. The aortic arch was separated and ligated with a No.27 needle using 7–0 nylon threads. Then the needle was removed to cause 60% annular coarctation of the aortic arch. After the operation, the chest of mice was closed, the wound was sutured. All animal experiments were performed according to the guideline of Guizhou Medical University. The study was approved by Guizhou Medical University.

### Animal grouping and treatment

The miRNA reagents, mir-125b agomir (agomir-125b) and scrambled control (NC agomir) were purchased from Shanghai GenePharma Co., Ltd. (Shanghai, China) and mixed with linear polyethyleneimine (PEI) nanoparticles (Sigma-Aldrich, St. Louis, MO) for intravenous injection. The construction of RNA interference plasmid of BAK1 (sh-BAK1) was completed by Shanghai Meixuan Biological Science and Technology Co., LTD. Empty pcDNA3.1 vector (Invitrogen, Shanghai, China) was used to construct BAK1 expression vector by Sangon (Shanghai, China). Sh-NC or empty pcDNA3.1 vector was used as the NC group.

Mice were randomly divided into ten groups (n = 8): (1) TAC group (mice were treated with TAC), (2) TAC + agomir-NC group (TAC mice were injected with agomir-125b negative control through the tail vein), (3) TAC + agomir-125b (TAC mice were injected with agomir-125b through the tail vein), (4) agomir-125b + sh-NC group (TAC mice were injected with agomir-125b and sh-NC through the tail vein), (5) agomir-125b + sh-BAK1 group (TAC mice were injected with agomir-125b and sh-BAK1 through the tail vein), (6) control group (mice without any treatment), (7) OE-BAK1 group (normal mice were injected with OE-BAK1 to overexpress BAK1), (8) OE-NC group (normal mice were injected with OE-NC), T9) AC + agomir-125b + OE-BAK1 (TAC mice were injected with agomir-125b and OE-BAK1), and (10) TAC + agomir-125b + OE-NC (TAC mice were injected with agomir-125b and OE-NC). For the sham mice, the steps were the same expecting the aortic arch was not ligated. Mice were sacrificed at 10 weeks of operation by intraperitoneal injection of excessive pentobarbital (100–150 mg/kg). The heart tissues of mice were harvested and used for the subsequent experiments. The sequences were 5’-UCCCUGAGACCCUAACUUGUGA-3’ for Agomir-125b, 5’-UUUGUACUACACAAAAGUACUG-3’ for Agomir-NC, 5’-AAAC GUAGCUUCGAAAGACCU for shBAK1, and 5’-GTTCTCCGAACGTGTCACGT-3’ for shNC.

### The detection of cardiac functions by ultrasound

The structure and function of mouse hearts were detected according to previous reports (Zhao et al. 2011; Jiang et al. 2014; Zhao et al. 2017) using the VeVo 770 high-resolution small animal ultrasound system. After mice were anesthetized by inhaling 2% isoflurane, the ultrasonic cardiogram of the limb leads of the mice was measured by using a 15 mm deep, 30 MHz probe. Two-dimensional guided M-mode tracings were recorded in both parasternal long and short axis views at the level of papillary muscles. The mean left ventricular ejection fraction (LVEF), left ventricular fractional shortening, left ventricular septal thickness, left ventricular septal diastole (IVS; d), left ventricular posterior wall thickness; left ventricular posterior diastole (LVPW; d), and left ventricular mass (LV mass) were calculated with the established standard equations. All measurements were made from at least three beats and averaged.

### H&E staining assay

The H&E staining assay was performed as previously described (Liu et al. 2018; Xu et al. 2019). Briefly, the heart tissues were collected, fixed with 4% paraformaldehyde at 4 °C, dehydrated with gradient alcohol, cleared in xylene, embedded, and sliced into 4 μm sections. Then the sections were dyed in hematoxylin, washed away with running water, and dyed with 1% eosin. After washing with running water again, the sections were dehydrated, cleared, blocked with neutral gum, and dried for three days. Finally, heart tissues were photographed under a light microscope in 200 × magnification. More than 100 myocytes in the sections were outlined in each group.

### Masson staining assay

The Masson staining assay was performed by using a Masson Stain Kit according to the instructions. The level of myocardial fibrosis was evaluated by calculating the percentage of myocardial tissue fibrosis or the blue region (collagen). For fibrosis measurements, > 25 fields were measured in each group.

### TUNEL staining assay

To evaluate the apoptosis rate of cardiomyocytes in mouse heart tissues, TUNEL staining was performed using the TUNEL BrightGreen Apoptosis Detection Kit according to the instructions. Of which, apoptotic nuclei were stained with green fluorescein and total cardiomyocyte nuclei were stained with DAPI. Representative images were captured using a confocal microscope and the apoptosis rate was calculated as TUNEL positive nuclei/DAPI-stained nuclei × 100%.

### Immunohistochemical staining assay

Immunohistochemistry staining was performed on deparaffinized sections to show collagen protein retention. After dewaxing and hydrating, the nonspecific antigens of the specimens were blocked with 3% H_2_O_2_ for 15 min and with 5% bovine serum albumin (BSA) (Beyotime, China) in phosphate buffer solution (PBS) for 45 min at 37 °C, respectively. Then, the specimens were incubated with primary antibodies against Col I (ab260043, 1:400, Abcam) and Col III (ab7778, 1:200, Abcam) at 4 °C for 12 h. After washed three times with PBS to remove the redundant primary antibodies, the specimens were incubated with tagged secondary goat anti-mouse lgG or goat anti-rabbit lgG (ZSGB-Bio, Beijing, China) at 37 °C for 60 min. Finally, the immunohistochemistry staining was stopped with DAB chromogen kit (ZSGB-BIO, Beijing, China) followed by hematoxylin staining, and the positive-staining was observed using a fluorescence microscope (Nikon, Japan).

### Immunofluorescent staining assay

Immunofluorescence staining was performed on deparaffinized sections. After dewaxed and hydrated, the nonspecific antigens of the specimens were blocked with 3% H_2_O_2_ for 15 min and with 5% bovine serum albumin (BSA) (Beyotime) in PBS for 45 min at 37 °C, respectively. Subsequently, the sections were incubated with primary anti-Cas-3 (ab32351, 1:200, Abcam) and anti-α-sarcomeric actin (ab68167, 1:200, Abcam) antibodies at 4 °C overnight followed by goat anti-rabbit IgG Alexa FluorVR 647 (ab150083, 1:1500, Abcam, Cambridge, United Kingdom). Nuclei were stained using DAPI (Beyotime, China). Angiogenesis was observed using a fluorescence microscope (Nikon, Japan).

### Extraction of primary cardiomyocytes

Mice were sacrificed at 10 weeks of operation by intraperitoneal injection of excessive pentobarbital (100–150 mg/kg) and immersed in 70% ethanol for 1 min. The hearts were removed and placed in PBS. The atrium and excess arterial tissues were cut off. Ventricular tissues were chopped and transferred into a 15 ml centrifuge tube. A disinfectant solution composed of 0.125% trypsin, 0.1% collagenase II and 0.05% DNase was added. After incubation at 37℃, the cell suspension was collected by centrifuge. The process was repeated for 3 times. The collected suspension was mixed with high glucose DMEM supplemented with 10% FBS, 1% penicillin and 1% streptomycin. After pipetting up and down several times, fibroblasts were filtered with a 230-mesh screen and separated using the differential attachment method at 60 min each time and placed at 37℃ in a three-gas incubator. Unattached primary cardiomyocytes were collected for Western blot.

### qRT-PCR

The total RNA was extracted from 60 mg heart tissues using the TRIzol reagent (Invitrogen, Carlsbad, USA) according to the instructions. After DNase I treatment, RNA was reverse transcribed into cDNA using with PrimeScript Reverse Transcriptase (TaKaRa, Japan) according to the manufacturer’s instruction and subjected to quantitative real-time PCR on LightCycler 480 (Roche, Basel, Switzerland). The relative expression changes of targets were analyzed by the 2^−ΔΔCT^ method, with GAPDH and U6 as the internal reference. The primers using in this study were mouse-ANP forward ACCACCTGGAGGAGAAGA and reverse TTCAAGAGGGCAGATC TATC, mouse-BNP forward TCCAGCAGAGACCTCAAAATTCC and reverse TCAAAGGTGGTCCCAGAGCT, mouse-β-MHC forward CCGAGTCCCAGGTCA ACAA and reverse CTTCACGGGCACCCTTGGA, mouse-GAPDH forward ACTCCACTCAC GGCAAATTC and reverse TCTCCATGGTGGTGAAGACA, miR-125b forward TCCCTGAGACCCTAACTTGTGA and reverse AGTCTCAGGGTCC GAGGTATTC, and U6 forward CTCGCTTCGGCAGCACA and reverse AACGC TTCACGAATTTGCGT.

### Western blot

Total proteins of heart tissues were isolated by using RIAP lysis buffer (Beyotime, China) according to the instructions, and protein concentrations were measured using the BCA Protein Assay Kit (Abcam, USA). Approximately equal amounts of proteins were separated by 12% SDS-PAGE and then transferred onto the PVDF membranes (Millipore, USA). After blocking by TBST containing 5% skim milk for 2 h at room temperature, the membranes were incubated with primary antibodies against BAK1 (Abcam, ab104124, 1:1000), C-caspase 3 (Abcam, ab2302, 1:300), Bax (Abcam, ab182733, 1:300) and Bcl-2 (Abcam, ab194583, 1:300) overnight at 4 °C, with GAPDH (Abcam, GAPDH,1:1000) as the internal reference. After washing, the membranes were exposed to horseradish peroxidase-labeled secondary antibody (Thermo Fisher Scientific, 1:200) for 2 h. Signals were detected by using the ECL kit and visualized by ChemiDoc™ MP System (Bio-Rad). The gray value of targets was analyzed with Image Lab™ Software. The protein expression level was normalized to GAPDH on the same PVDF membrane.

### Luciferase reporter assay

To determine the correlation between miR-125b and BAK1, the fragments containing wild type (WT) or mutant type (MUT) 3’ UTR of BAK1 with the putative miR-125b binding sites were amplified and cloned into pmiR-RB-Report vector. The luciferase reporter plasmids were co-transfected with miR-125b mimics or miR-NC into HEK293T cells using Lipofectamine 2000 according to the manufacturer’s instructions. After transfection for 48 h, the relative Renilla luciferase activities were measured using Dual-Luciferase Reporter Assay System.

### Statistical analysis

Data were analyzed using GraphPad Prism 7.0 and presented as mean ± standard deviation (SD). Each experiment was repeated at least three times. After verifying data normality, the difference between two groups was determined by using the t test and among multiple groups was determined by one-way ANOVA in SPSS 19.0. P < 0.05 was considered significant.

## Results

### MiR-125b was significantly downregulated in heart tissues of HF mice

**Fig. 1:**
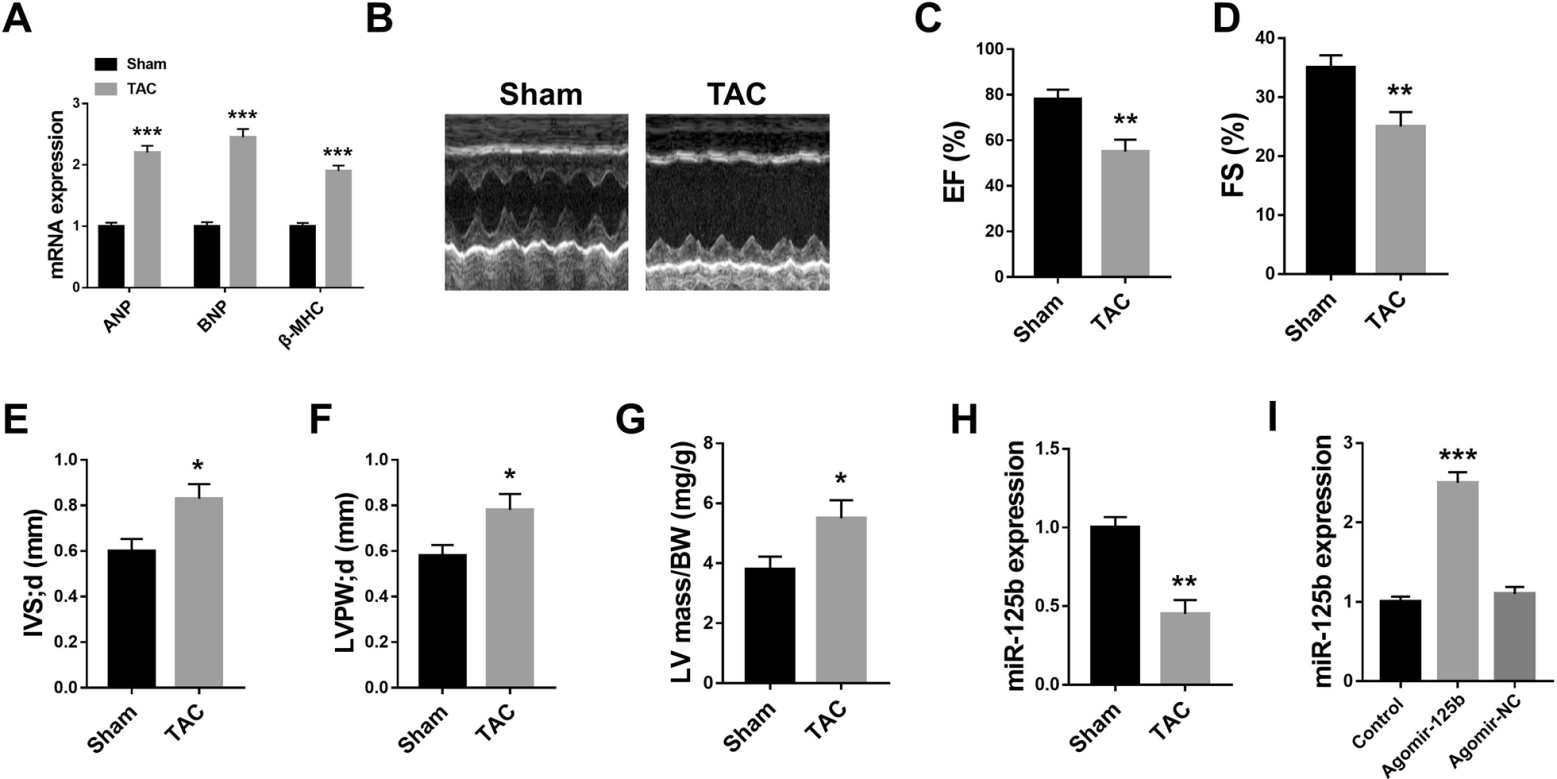
MiR-125b was downregulated in the heart tissues of HF mice. Mice were treated with or without TAC, and the structure and function of mice hearts were evaluated ten weeks after TAC operation by high resolution small animal ultrasound system and analyzed by M mode. **A** The mRNA levels of ANP, BNP and β-MHC were evaluated by qRT-PCR (n = 5). **B** Representative images of echocardiographic measurements of mice hearts (n = 8). **C** Left ventricular ejection fraction (EF%) (n = 8). **D** Left ventricular fractional shortening (FS%) (n = 8). **E** Left ventricular septal thickness; diastole (IVS; d) (n = 8). **F** Left ventricular posterior wall thickness; diastole, (LVPW; d) (n = 8). **G** Left ventricular mass/body weight (LV mass/BW) (n = 8). **H** The mRNA level of miR-125b was evaluated by qRT-PCR (n = 5). **I** Agomir-125b or agomir-NC was injected into mice, and the mRNA level of miR-125b was evaluated by qRT-PCR (n = 5). ^*^ p < 0.05, ^**^ p < 0.01, ^***^ p < 0.001 vs. sham group or control group

First, the HF mice model was established through TAC induction, and the heart function was evaluated ten weeks after the operation. The results showed that the mRNA expression of ANP, BNP and β-MHC was markedly upregulated in the heart tissues of TAC mice compared with that of the sham group (p < 0.001) (Fig. 1A). Meanwhile, compared with the sham group, the left ventricular ejection fraction (p < 0.01) (Fig. 1B and C) and left ventricular fractional shortening (p < 0.01) (Fig. 1D) was significantly decreased in the heart tissues of TAC mice. By contrast, left ventricular septal thickness diastole (IVS; d) (p < 0.05) (Fig. 1E) and left ventricular posterior wall thickness diastole (LVPW; d) (p < 0.05) (Fig. 1F) were increased in the heart tissues of TAC mice compared with those of the sham group. Similarly, compared with the sham group, the ratio of left ventricular mass/body weight (LV mass/BW) was also increased in the heart tissues of TAC mice (p < 0.05) (Fig. 1G). These data suggested that the HF mice model was successfully established ten weeks after TAC operation and could be used for the subsequent analysis. Then miR-125b expression was evaluated, and the results indicated that miR-125b mRNA level was significantly downregulated in the heart tissues of TAC mice in comparison with the sham group (p < 0.01) (Fig. 1H). Further, agomir-125b or agomir-NC was applied through the tail vein to overexpress miR-125b, and the efficiency was evaluated by qRT-PCR assay. The results showed that agomir-125b significantly increased miR-125b expression compared with the agomir-NC group (p < 0.001) (Fig. 1I). These results suggested that miR-125b plays a potential protective role for cardiac function during heart failure.

### MiR-125b overexpression alleviated cardiac function injury in HF mice

**Fig. 2:**
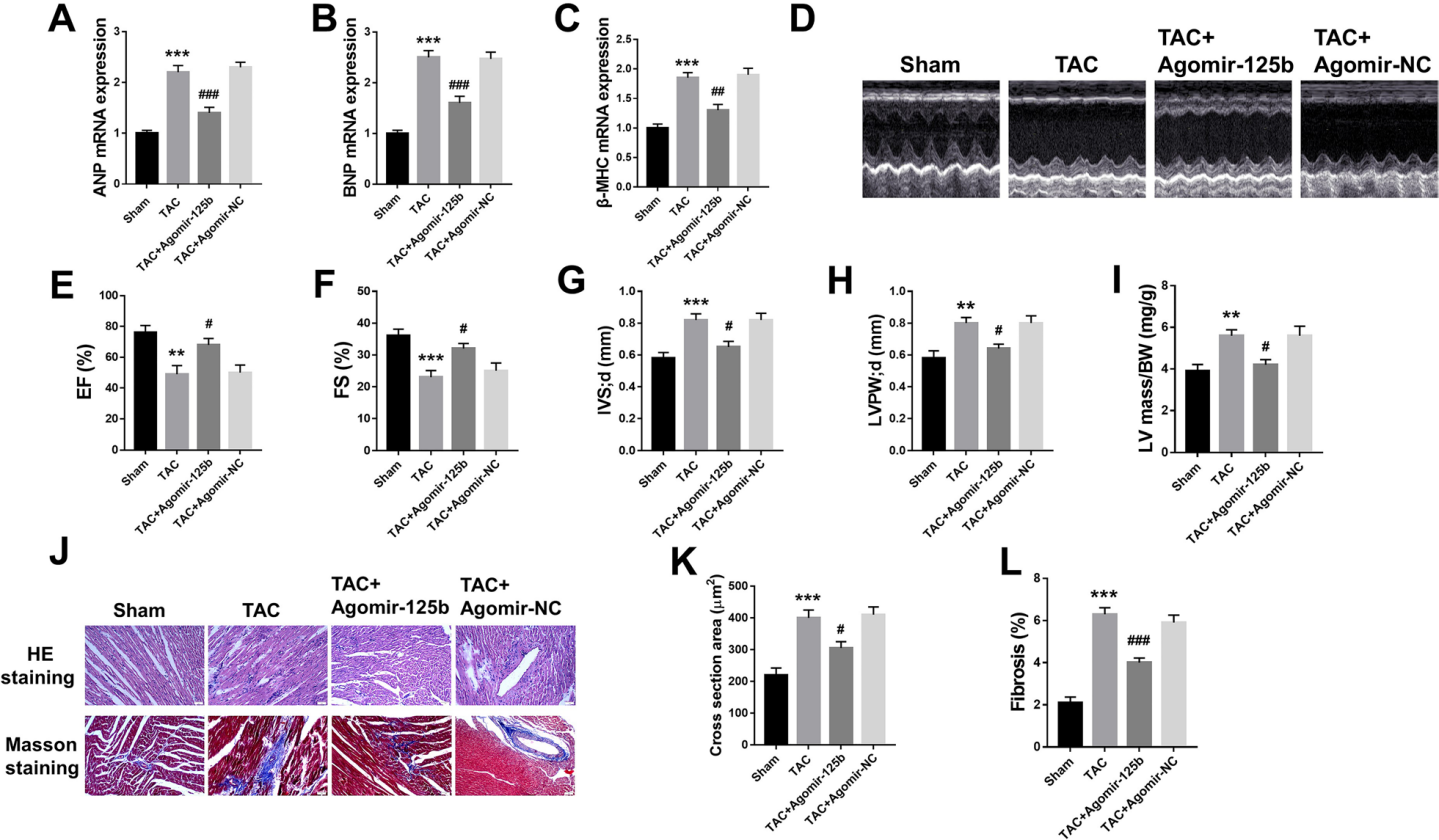
MiR-125b overexpression alleviated cardiac function injury in HF mice. Mice were treated with or without TAC, then TAC mice were tail intravenously injected with agomir-125b or agomir-NC. **A**–**C** The mRNA levels of ANP (**A**), BNP (**B**) and β-MHC (**C**) were evaluated by qRT-PCR (n = 5). **D** Representative images of echocardiographic measurements of mouse hearts (n = 8). **E** Left ventricular ejection fraction (EF%) (n = 8). **F** Left ventricular fractional shortening (FS%) (n = 8). **G** Left ventricular septal thickness; diastole (IVS; d) (n = 8). **H** Left ventricular posterior wall thickness; diastole, (LVPW; d) (n = 8). **I** Left ventricular mass/body weight (LV mass/BW) (n = 8). **J** Representative images of H&E staining and Masson staining of mouse heart tissues (n = 8). **K** The statistical result of H&E staining (n = 5). **L** The statistical result of Masson staining (n = 5). ^*^ p < 0.05, ^**^ p < 0.01, ^***^ p < 0.001 vs. sham group; ^#^ p < 0.05, ^##^ p < 0.01, ^###^ p < 0.001 vs. TAC group

To determine the role of miR-125b in heart tissues, agomir-125b was intravenously injected through tail into TAC mice to overexpress miR-125b. Compared with TAC mice, the mRNA expression of ANP (p < 0.001) (Fig. 2A), BNP (p < 0.001) (Fig. 2B) and β-MHC (p < 0.01) (Fig. 2C) in the heart tissues was all significantly decreased in TAC + agomir-125b group, while exhibited no obvious change in TAC + agomir-NC agomir-125b group. Meanwhile, compared with TAC group, the left ventricular ejection fraction (p < 0.05) (Fig. 2D and E) and left ventricular fractional shortening (p < 0.05) (Fig. 2F) were increased, while left ventricular septal thickness diastole (IVS; d) (p < 0.05) (Fig. 2G), left ventricular posterior wall thickness diastole (LVPW; d) (p < 0.05) (Fig. 2H) and left ventricular mass/body weight (LV mass/BW) (p < 0.05) (Fig. 2I) were decreased in TAC + agomir-125 group and showed no obvious change in TAC + agomir-NC group. Moreover, H&E staining and Masson staining (Fig. 2J) were performed to evaluate the degree of myocardial damage. The data indicated that TAC treatment significantly increased the percentage of cross section in heart tissues compared with the sham group (p < 0.001), while miR-125b overexpression significantly decreased the effect of TAC effect on cross section (p < 0.01) (Fig. 2K). Furthermore, TAC treatment significantly increased the percentage of myocardial tissue fibrosis compared with the sham group (p < 0.001), while miR-125b overexpression significantly decreased the effect of TAC on myocardial tissue fibrosis (p < 0.001) (Fig. 2L). Additionally, about 80% of the myocardial collagen fibers were Col I, which can maintain the strength of the ventricular wall due to its great hardness and strong anti-pull function, and about 11% were Col III, which has relatively fine fibrosis and good extensibility and elasticity (Bodh et al. 1995; Hanna et al. 2021). The ratio of these two collagens is of great significance for maintaining the normal structure of cardiac tissue and the integrity of cardiac function (Bodh et al. 1995; Hanna et al. 2021). We then demonstrated that miR-125b overexpression alleviated Col I expression in TAC-induced myocardial tissues and promoted Col III expression (Additional file 1: Fig. S1). These results indicated that miR-125b overexpression efficiently attenuated TAC-induced cardiac function injury.

### MiR-125b overexpression inhibited cardiomyocyte apoptosis in HF mice

**Fig. 3:**
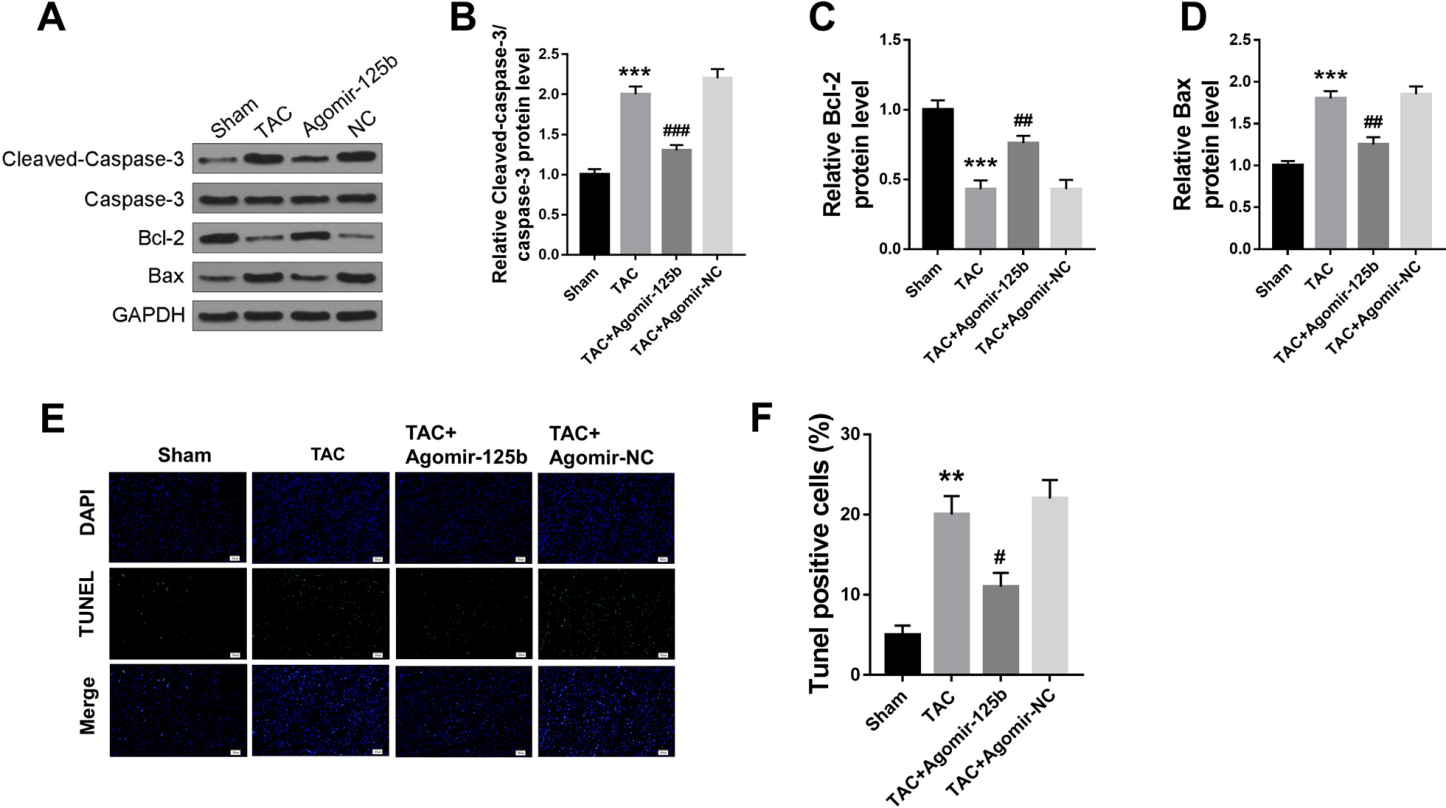
MiR-125b overexpression inhibited cardiomyocyte apoptosis in HF mice. Mice were treated with or without TAC. TAC mice were tail intravenously injected with agomir-125b or agomir-NC. **A** The protein expression of C-caspase 3, caspase 3, Bcl-2 and Bax in heart tissues was detected by Western blot (n = 5). **B**–**D** The relative expression changes in C-caspase 3 (**B**), Bcl-2 (**C**) and Bax (**D**) were analyzed by Image Lab™ Software (n = 5). **E** Representative images of TUNEL staining of heart tissues by confocal microscopy (n = 8). **F** The apoptosis rate was calculated as the ratio of TUNEL positive nuclei to DAPI-stained nuclei (n = 5). ^*^ p < 0.05, ^**^ p < 0.01, ^***^ p < 0.001 vs. sham group; ^#^ p < 0.05, ^##^ p < 0.01, ^###^ p < 0.001 vs. TAC group

To explore whether miR-125b affects the heart failure through modulating the process of cardiomyocyte apoptosis, the expression of apoptosis-related proteins in heart tissues was detected by Western blot. The results showed that TAC treatment significantly increased the protein expression of C-caspase 3 (p < 0.001) (Fig. 3A and B) and Bax (p < 0.001) (Fig. 3A and D) compared with the sham group and decreased Bcl-2 expression (p < 0.001) (Fig. 3A and C), while miR-125b overexpression significantly reversed the effect of TAC operation on the expression of C-caspase 3, Bcl-2 and Bax (p < 0.01, p < 0.001) (Fig. 3A–D). Meanwhile, the apoptosis of cardiomyocyte was evaluated by TUNEL staining (Fig. 3E), and the results indicated that TAC treatment significantly increased the percentage of TUNEL positive cells compared with the sham group (p < 0.01), while miR-125b overexpression significantly decreased the percentage of TAC-induced TUNEL positive cells compared with agomir-NC group (p < 0.05) (Fig. 3F). To determine the origin of these apoptotic markers from cardiomyocytes, we performed immunofluorescence staining (Additional file 2: Fig. S2A) and Western blot (Additional file 2: Fig. S2B) fto examined the levels of both Cas-3 and myocyte marker α-Sarcomeric actin. The results revealed that miR-125b overexpression efficiently inhibited cardiomyocyte apoptosis in HF mice.

### BAK1 was a target of miR-125b

**Fig. 4:**
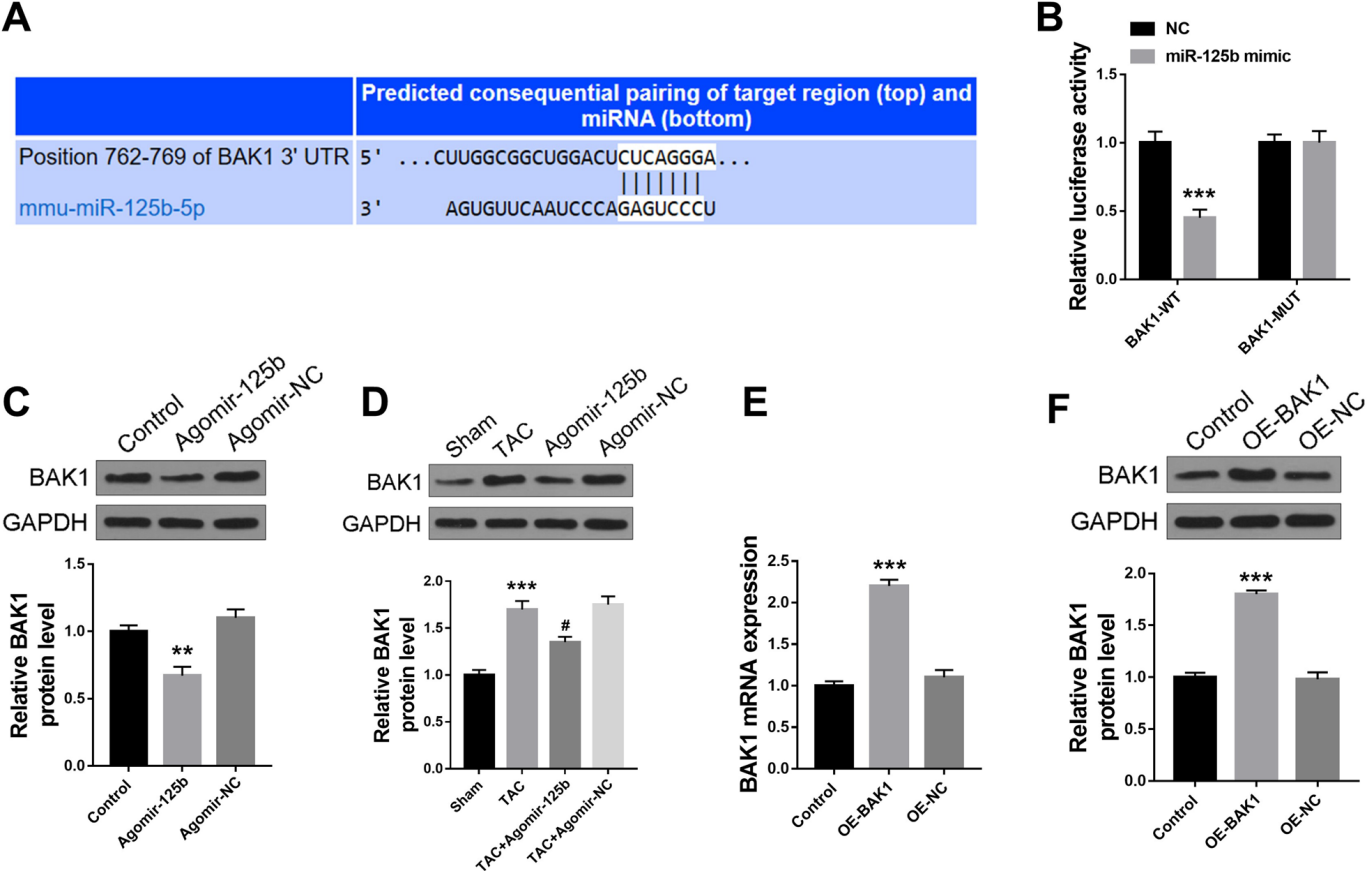
BAK1 was a target of miR-125b. **A** The putative binding sites between miR-125b and 3′ UTR of BAK1 was predicted by Targetscan database. **B** The relative luciferase activity in HEK293T cells was evaluated by dual luciferase report system (n = 6). ^***^ p < 0.001 vs. miR-NC group. **C** Agomir-125b or agomir-NC was tail intravenously injected into normal mice, and the protein expression of BAK1 was evaluated by Western blot (n = 5). ^**^ p < 0.01, ^***^ p < 0.001 vs. control group. **D** Agomir-125b or agomir-NC was tail intravenously injected into TAC mice, and the protein expression of BAK1 was evaluated by Western blot (n = 5). ^**^ p < 0.01, ^***^ p < 0.001 vs. sham group. **E** and **F** OE-BAK1 or OE-NC was tail intravenously injected into normal mice, then mRNA expression (**E**) and protein expression (**F**) was evaluated (n = 5). ^***^ p < 0.001 vs. control group

Next, the Targetscan database was used to predict the potential targets of miR-125b, and the results showed that there was a putative binding site between miR-125b and 3′ UTR of BAK1 (Fig. 4A). Then the fragments containing WT or MUT 3′ UTR of BAK1 were co-transfected with miR-125b mimics or miR-NC into HEK293T cells, and the luciferase reporter assay was performed. The results indicated that miR-125b mimics significantly decreased the relative luciferase activity of WT 3′ UTR of BAK1 (p < 0.001), while showed no obvious change of the MUT 3′ UTR of BAK1 (Fig. 4B). Further, agomir-125b or agomir-NC was tail intravenously injected into normal mice, and the results indicated that miR-125b overexpression significantly decreased BAK1 protein level in heart tissues compared with the agomir-NC group (p < 0.01) (Fig. 4C). In addition, agomir-125b or agomir-NC was tail intravenously injected into TAC mice, and the results indicated that TAC treatment markedly increased BAK1 protein level compared with the sham group (p < 0.001), and miR-125b overexpression observably decreased TAC-induced BAK1 expression compared with the agomir-NC group (p < 0.05) (Fig. 4D). Moreover, BAK1 overexpression vector (OE-BAK1) or negative control (OE-NC) was constructed and tail intravenously injected into normal mice, and the results indicated that BAK1 overexpression significantly increased BAK1 expression at both mRNA (p < 0.001) (Fig. 4E) and protein (p < 0.001) (Fig. 4F) levels in heart tissues of mice compared with the OE-NC group. These results suggested that BAK1 was a direct target of miR-125b.

### BAK1 overexpression significantly reversed agomir-125b-induced protective effects in heart failure

**Fig. 5:**
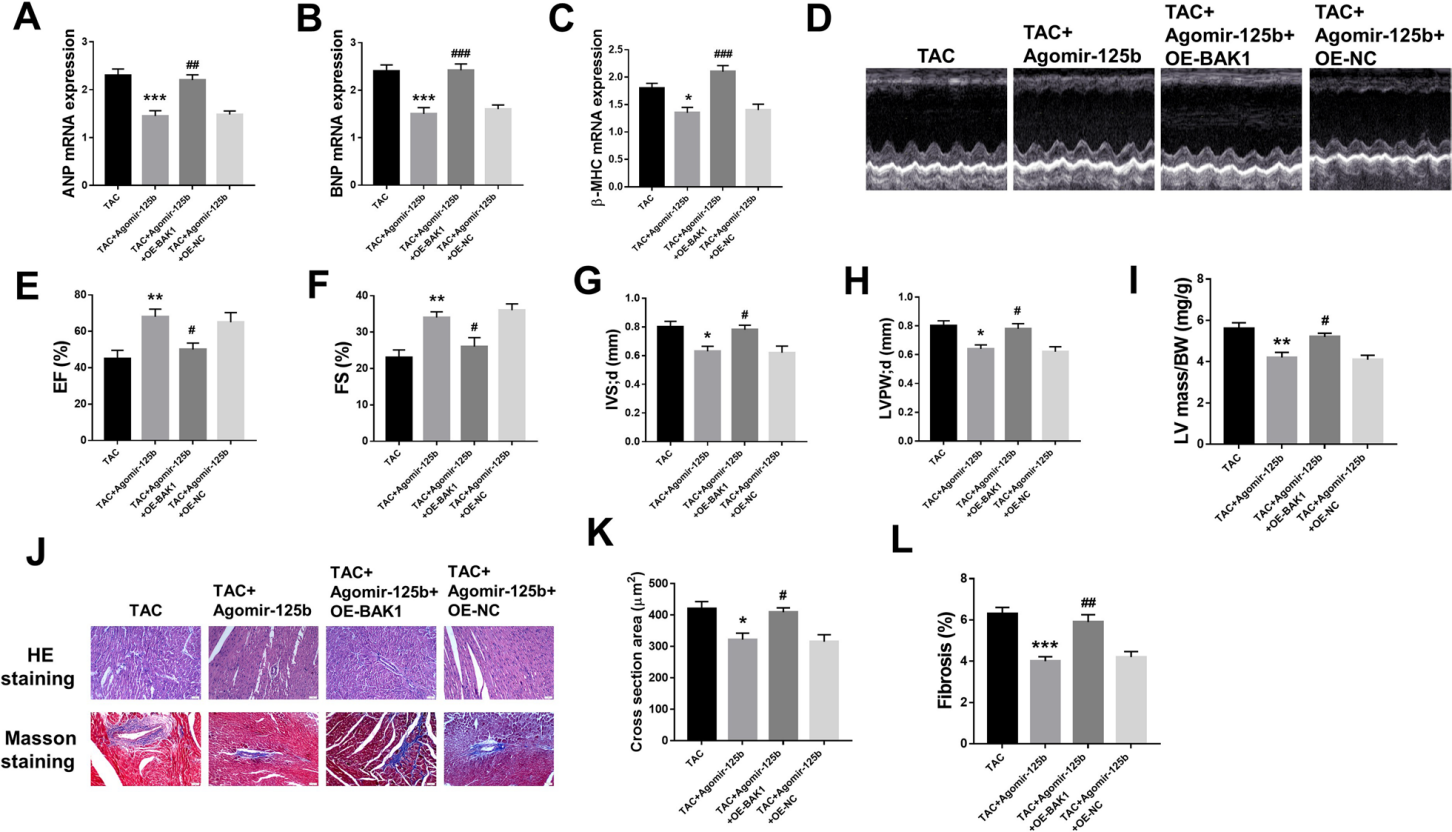
BAK1 overexpression significantly reversed agomir-125b-induced protective effect in heart failure. TAC mice were transfected with agomor-125b, or co-transfected with agomir-125b and OE-BAK1, or agomir-125b and OE-NC. (A-C) The mRNA level of ANP (**A**), BNP (**B**) and β-MHC (**C**) was evaluated by qRT-PCR (n = 5). **D** Representative images of echocardiographic measurements of mice hearts (n = 8). **E** Left ventricular ejection fraction (EF%) (n = 8). **F** Left ventricular fractional shortening (FS%) (n = 8). **G** Left ventricular septal thickness; diastole (IVS; d) (n = 8). **H** Left ventricular posterior wall thickness; diastole, (LVPW; d) (n = 8). **I** Left ventricular mass/body weight (LV mass/BW) (n = 8). **J** Representative images of H&E staining and Masson staining of mouse heart tissues (n = 8). **K** The statistical result of H&E staining (n = 5). **L** The statistical result of Masson staining (n = 5). * p < 0.05, ** p < 0.01, *** p < 0.001 vs. TAC group; ^##^ p < 0.01, ^###^ p < 0.001 vs. TAC + agomir-125b group

To determine whether the protective effect of miR-125b overexpression was mediated by BAK1, TAC mice were co-transfected with agomir-125b and OE-BAK1, or with agomir-125b and OE-NC, and the heart function was evaluated. Co-transfection with agomir-125b and OE-BAK1 significantly reversed agomir-125b-induced protective effect in TAC mice on the mRNA level of ANP (p < 0.01) (Fig. 5A), BNP (p < 0.001) (Fig. 5B) and β-MHC (p < 0.001) (Fig. 5C), the left ventricular ejection fraction (p < 0.05) (Fig. 5D and E), left ventricular fractional shortening (p < 0.05) (Fig. 5F), left ventricular septal thickness diastole (IVS; d) (p < 0.05) (Fig. 5G), left ventricular posterior wall thickness diastole (LVPW; d) (p < 0.05) (Fig. 5H) and left ventricular mass/body weight (LV mass/BW) (p < 0.05) (Fig. 5I) compared with the agomir-125b and OE-NC group. In addition, the H&E staining and Masson staining (Fig. 5J) were performed to evaluate the degree of myocardial damage. The results indicated that co-transfection with agomir-125b and OE-BAK1 significantly increased the percentage of cross section (p < 0.05) (Fig. 5K) and myocardial tissue fibrosis (p < 0.01) (Fig. 5L) in heart tissues compared with the agomir-125b and OE-NC group. We also demonstrated that OE-BAK1 overexpression reversed the effect of mir-125b on Col I and col III expression of TAC-induced myocardial tissue (Additional file 3: Fig. S3). These results demonstrated that BAK1 overexpression significantly reversed agomir-125b-induced protective effect in heart failure.

### BAK1 overexpression significantly reversed agomir-125b-inhibited cardiomyocyte apoptosis in HF mice

**Fig. 6:**
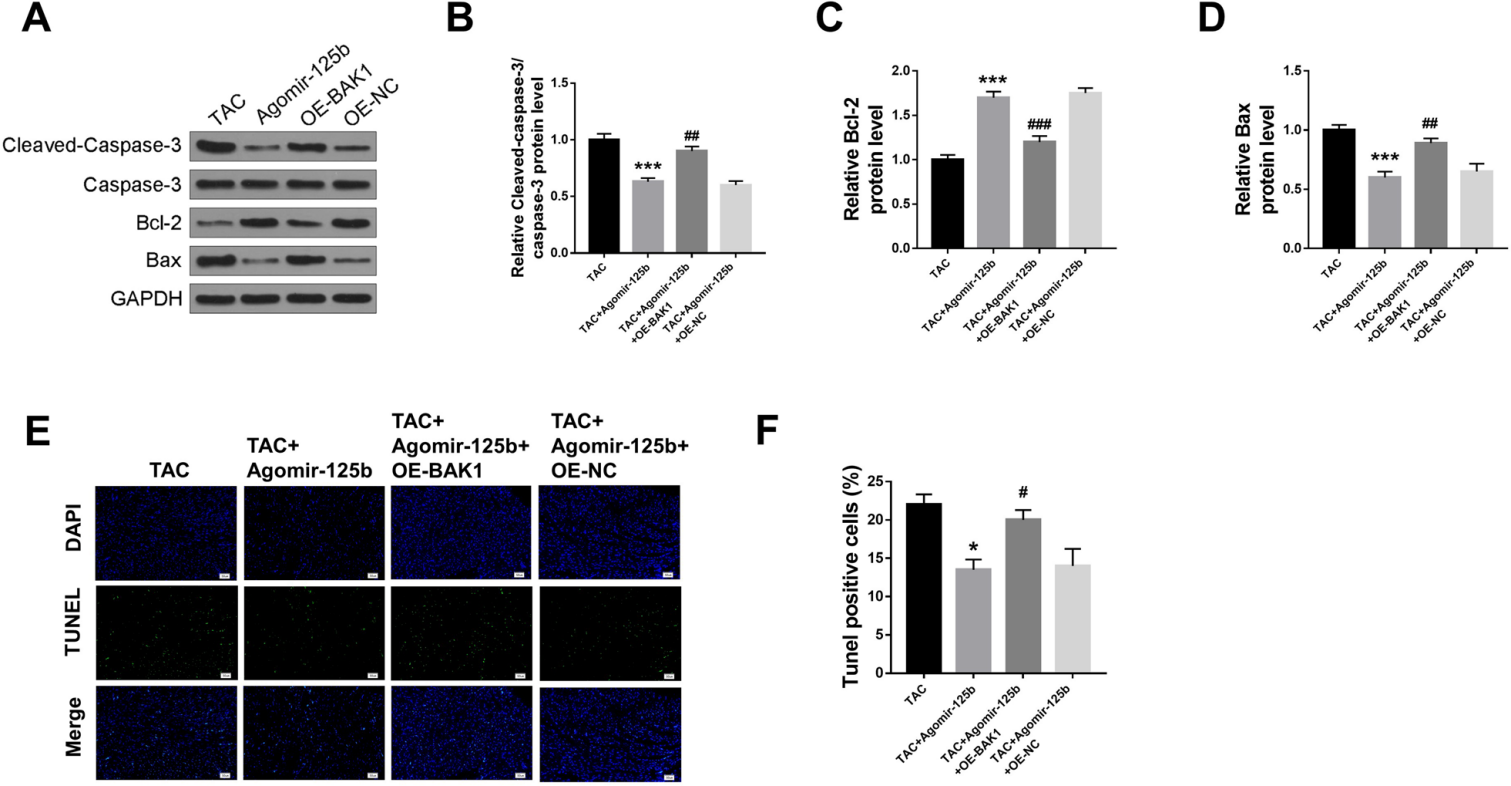
BAK1 overexpression significantly reversed agomir-125b-inhibited cardiomyocyte apoptosis in HF mice. TAC mice were co-transfected with agomir-125b and OE-BAK1, or agomir-125b and OE-NC. **A** The protein expression of C-caspase 3, caspase 3, Bcl-2 and Bax in heart tissues was detected by western blot (n = 5). **B**–**D** The relative expression changes in C-caspase 3 (**B**), Bcl-2 (**C**) and Bax (**D**) were analyzed by Image Lab™ Software (n = 5). **E** Representative images of TUNEL staining of heart tissues by confocal microscopy (n = 8). **F** The apoptosis rate was calculated as the ratio of TUNEL positive nuclei to DAPI-stained nuclei (n = 5). * p < 0.05, ** p < 0.01, *** p < 0.001 vs. TAC group; # p < 0.05, ## p < 0.01, ### p < 0.001 vs. TAC + agomir-125b group

TAC mice were co-transfected with agomir-125b and OE-BAK1, or with agomir-125b and OE-NC, and the index of myocardial apoptosis was evaluated. The results indicated that co-transfection with agomir-125b and OE-BAK1 significantly increased the protein expression of C-caspase 3 (p < 0.01) (Fig. 6A and B) and Bax (p < 0.01) (Fig. 6A and D) induced by agomir-125b, while decreased Bcl-2 expression (p < 0.001) (Fig. 6A and B) compared with the agomir-125b and OE-NC group. In addition, cardiomyocyte apoptosis was evaluated by TUNEL staining (Fig. 6E), and the results showed that co-transfection with agomir-125b and OE-BAK1 significantly increased the percentage of TUNEL positive cardiomyocytes with miR-125b overexpression compared to those with agomir-125b and OE-NC overexpression (p < 0.05) (Fig. 6F). We also further identified that apoptosis markers were derived from cardiomyocytes (Additional file 4: Fig. S4). These results revealed that BAK1 overexpression significantly reversed agomir-125b inhibited-cardiomyocyte apoptosis in HF mice.

## Discussion

HF is increasing in prevalence with a lack of efficient therapies that produce major severe effects on its associated mortality (McMurray and Pfeffer 2005). Recently, a large number of microRNAs have been identified to be closely associated with the progression of HF and might be considered as the potential targets for therapy (Shah et al. 2017; Qiao et al. 2019; Verjans et al. 1979). Wang et al. found that the heart-related circRNA (HRCR) protects the heart against pathological hypertrophy and HF through directly targeting miR-223 (Wang et al. 2016). Yang et al. found that miR-19b-1 reverses ischemia-induced heart failure through inhibiting cardiomyocyte apoptosis and targeting Bcl2-like 11/BIM (a pro-apoptotic gene of the Bcl-2 family) (Yang et al. 2019). Although miR-125b exerts broad effects in human diseases, the specific function of miR-125b in HF remains unclear and attracts our attention. In the present study, to explore the role and underlying mechanisms of miR-125b in detail, the HF mice model was successfully established by TAC operation. Interestingly, our results indicated that miR-125b was significantly downregulated in the heart tissues of HF mice, suggesting a potential protective effect of miR-125b in HF.

Myocardial interstitial fibrosis is well known to result in left ventricular dysfunction, contributing to the development of HF (González et al. 2018). It has been reported that myocardial interstitial fibrosis is a major cause of cardiac dysfunction with adverse tissue remodeling (Schelbert et al. 2014; Butler et al. 2014; Davis and Molkentin 2014). Current therapies that improve HF survival primarily target the pathogenic mechanisms that occur within cardiomyocytes but not those that take place outside cardiomyocytes in the interstitial space that houses collagen and fibroblasts (Yang et al. 2016). This lack of treatment against interstitial mechanisms of HF might be one cause of mortality and morbidity for HF. Here, our results demonstrated that miR-125b overexpression efficiently inhibited TAC-induced myocardial tissue fibrosis. In addition, cardiomyocyte apoptosis also plays an important facilitation effect in the progression of HF. Inhibition of cardiomyocyte apoptosis can efficiently protect the heart against HF (Deng et al. 2019; Li et al. 2018b). Apoptosis-related markers such as C-caspase 3, Bax and Bcl-2 are often used to represent the degree of cell apoptosis, of which, caspases (caspase-3, caspase-6, and caspase-8) are a family of cysteine proteases that mediate apoptosis induced by a variety of stimuli (Jin et al. 2009), Bax and Bcl-2 are the members of anti-apoptotic Bcl-2 family, and Bax is a pro-apoptotic factor (Edlich 2018). Our results further demonstrated that miR-125b overexpression efficiently attenuated TAC-induced cardiac function injury, including myocardial tissue fibrosis. Meanwhile, miR-125b overexpression significantly reversed the TAC-induced effect on the expression of apoptosis-related proteins including C-caspase 3, Bax and Bcl-2, suggesting that miR-125b overexpression significantly inhibited TAC-induced cardiomyocyte apoptosis in HF mice.

BAK1, a pro-apoptotic factor, has been reported to act as the direct target of miRNAs, is involved in cardiac apoptosis and MI (Su et al. 2020; Zhou et al. 2019). In the heart, BAK1 was reported to induce cardiomyocyte and myocardial I/R-mediated apoptosis (Bayoumi et al. 2018b). Recent studies have shown that BAK1 was regulated by miR-125b in cancer cells and neural crest cells (Wang et al. 2013; Chen et al. 2015). It has also been demonstrated that miR-125b-5p protected the heart from AMI by repressing BAK1 expression in cardiomyocytes. In our study, the bioinformatic analysis showed that there were complementary bases between miR-125b and BAK1. Moreover, luciferase analysis and miR-125b overexpression assay also showed that BAK1 was a direct target of miR-26a. Reportedly, BAK1 was significantly increased in mouse hearts during I/R injury and in cardiomyocytes subjected to I/R simulation (Bayoumi et al. 2018b). We also demonstrated that BAK1 expression in TAC-induced HF mice was significantly upregulated. Moreover, BAK1 overexpression significantly reversed agomir-125b-induced protective effect on cardiac function injury, cardiomyocyte apoptosis and the expression of apoptosis-related markers C-caspase3, Bax and Bcl-2.

## Conclusion

In conclusion, our results demonstrated that miR-125b overexpression efficiently alleviated HF through inhibiting cardiomyocyte apoptosis by targeting BAK1, suggesting that miR-125b/BAK1 axis might be a potential target for the treatment of HF.

## Supplementary Information

**Additional file 1: Fig. S1.** MiR-125b overexpression alleviated the expression of Col I and promoted col III expression in TAC-induced myocardial tissues. * p < 0.05.

**Additional file 2: Fig. S2**. MiR-125b overexpression efficiently inhibited cardiomyocyte apoptosis in HF mice. Immunofluorescence staining (A) and Western Blot of isolated myocytes (B) for both Cas-3 and myocyte markers were performed to conclude that these apoptotic markers were from cardiac myocytes. * p < 0.05.

**Additional file 3: Fig. S3**. BAK1 overexpression reversed the effects of mir-125b on Col I and col III expression in TAC-induced myocardial tissues. * p < 0.05.

**Additional file 4: Fig. S4.** BAK1 overexpression significantly reversed agomir-125b-inhibited cardiomyocyte apoptosis in HF mice. Immunofluorescence staining (A) and Western Blot of isolated myocytes (B) for both Cas-3 and myocyte marker were performed to conclude that these apoptotic markers came from cardiac myocytes. * p < 0.05.

## Data Availability

The data that support the findings of this study are available on request from the Corresponding authors. The data are not publicly available due to their containing information that could compromise the privacy of research participants.
