# Supplementary figures and images for: MiR-125b inhibits cardiomyocyte apoptosis by targeting BAK1 in heart failure
Source: Mol Med. 2021 Jul 8;27:72. doi: 10.1186/s10020-021-00328-w (PMC8268255; doi:10.1186/s10020-021-00328-w)

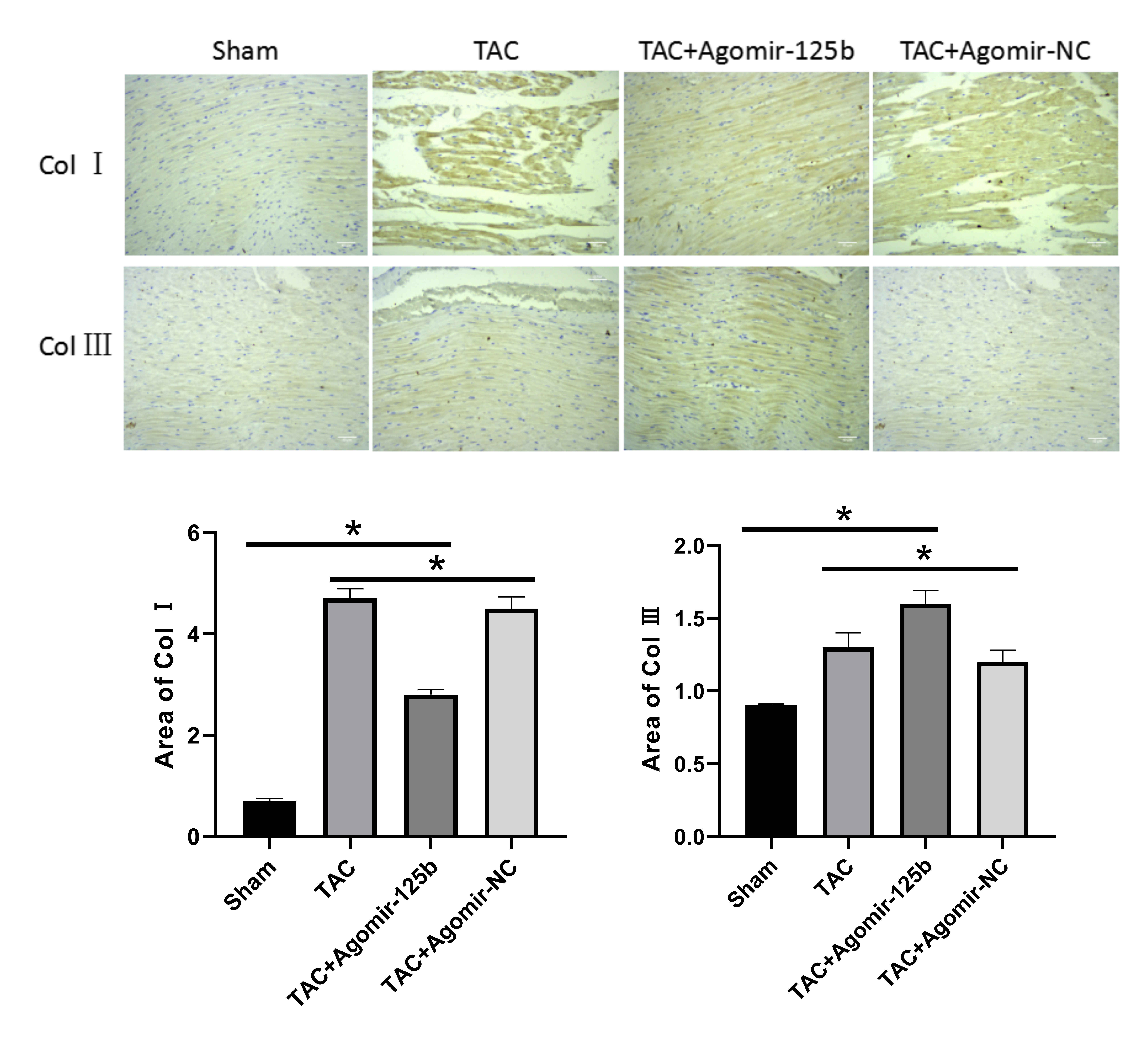

Supplement: Supplementary file 1 — Additional file 1: Fig. S1. MiR-125b overexpression alleviated the expression of Col I and promoted col III expression in TAC-induced myocardial tissues. * p < 0.05. [file 10020_2021_328_MOESM1_ESM.tif]

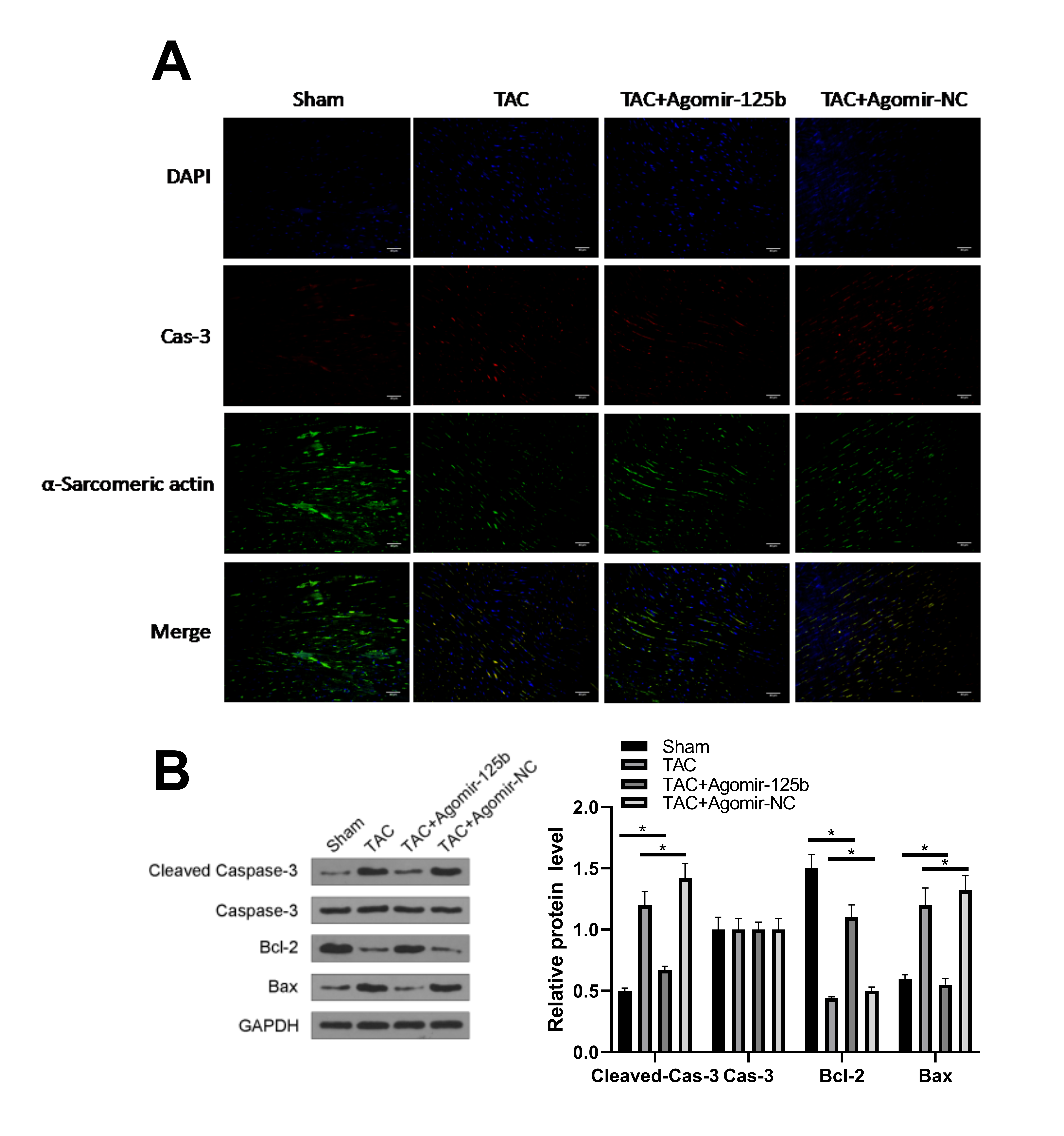

Supplement: Supplementary file 2 — Additional file 2: Fig. S2. MiR-125b overexpression efficiently inhibited cardiomyocyte apoptosis in HF mice. Immunofluorescence staining (A) and Western Blot of isolated myocytes (B) for both Cas-3 and myocyte markers were performed to conclude that these apoptotic markers were from cardiac myocytes. * p < 0.05. [file 10020_2021_328_MOESM2_ESM.tif]
